# Supplementary material for: Imaging and controlling plasmonic interference fields at buried interfaces
Source: Nat Commun. 2016 Oct 11;7:13156. doi: 10.1038/ncomms13156 (PMC5062594; doi:10.1038/ncomms13156)
Supplement: Supplementary Information — Supplementary Figures 1-8 and Supplementary References. [file ncomms13156-s1.pdf]

Supplementary Figures

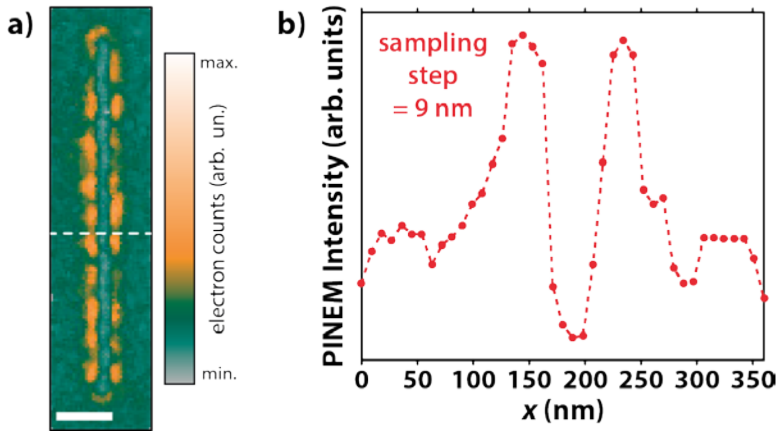

Supplementary Figure 1: Spatial resolution of time-resolved PINEM imaging. (a) Experimental photon-induced near-field electron microscopy (PINEM) image[1] of the surface plasmon polariton (SPP) field distribution on an isolated silver nanowire (3.3 μm length, 45 nm radius), photoinduced with pulsed light excitation polarized parallel to the wire long axis (800 nm). The image was recorded at $\tau = 0$ fs, using only electrons that have gained energy. The horizontal dashed line corresponds to the cross-section data plotted in panel b. The scale bar corresponds to 200 nm. **(b)** Cross-section of the experimental image in panel a, showing the sharp left edge of the silver nanowire being within one spatial sampling step of 9 nm.

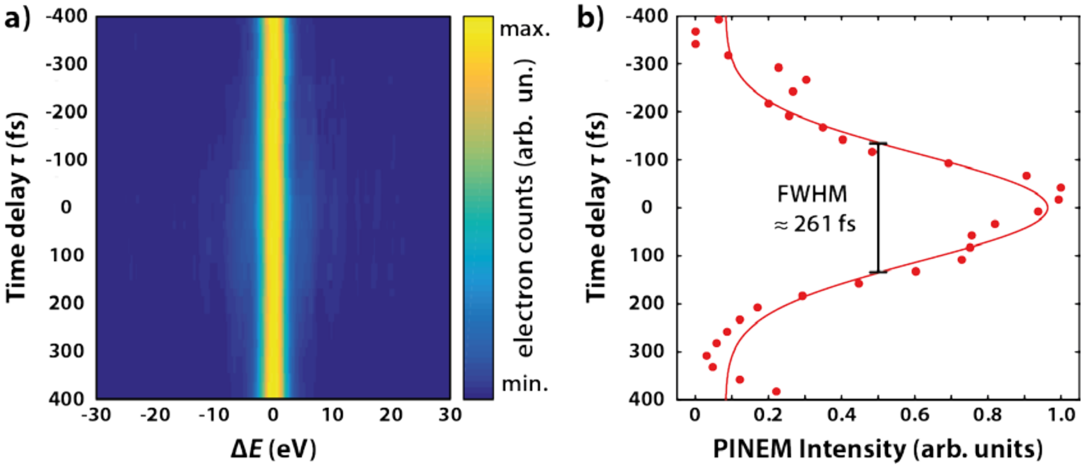

Supplementary Figure 2: Current temporal resolution of time-resolved PINEM experiments. (a) Map of the electron energy loss (EEL) spectrum versus relative time delay τ, taken on a single photoexcited silver nanowire (800 nm pulsed excitation light, polarized at 45 degrees to the wire long axis, pulse duration ≈ 57 fs). The temporal reference is set by choosing the relative time delay τ to be zero at the maximum photon-induced near-field electron microscopy (PINEM) intensity. The color map indicates the linear intensity map in units of electron counts. **(b)** Corresponding temporal cross-correlation (data points), obtained after removal of the zero-loss peak contributions by subtraction of a Gaussian line profile fitted to experimental spectra at negative delay. A Gaussian fit to the data (solid line) yields a full-width-at-half-maximum (FWHM) of ≈ 261 fs, which after deconvolution from the optical excitation pulse (Gaussian FWHM ≈ 60 fs) and a 100 fs plasmon lifetime yields an estimated electron bunch duration of ≈ 196 fs.

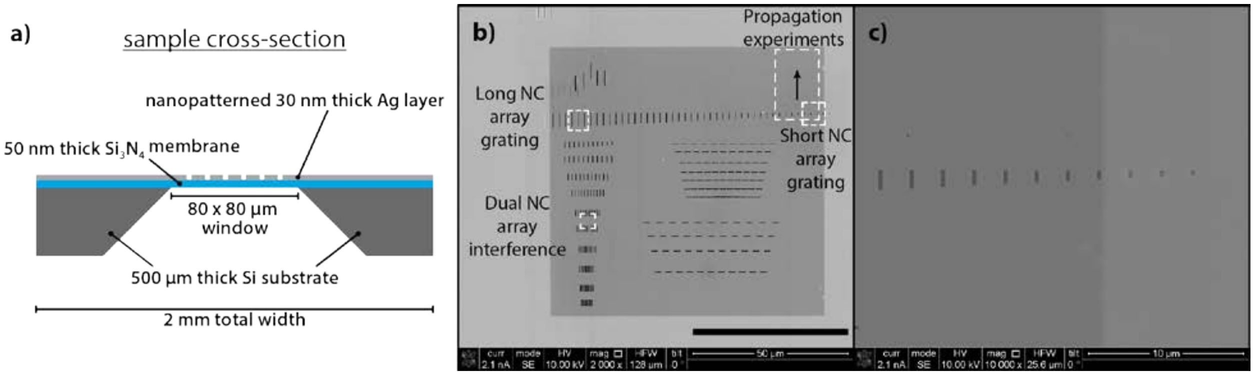

Supplementary Figure 3: The nanopatterned Ag-on-Si_3N_4 sample. (a) Schematic cross-section of the sample. **(b)** Overview scanning electron microscope (SEM) image of the various perforating nanocavities written in the Ag film. Areas imaged using the photon-induced near-field electron microscopy (PINEM) technique in this work are indicated by labeled dashed rectangles. **(c)** SEM zoom-in on the nanocavity array imaged in the propagation experiment described in figures 2 and 3 of the main text.

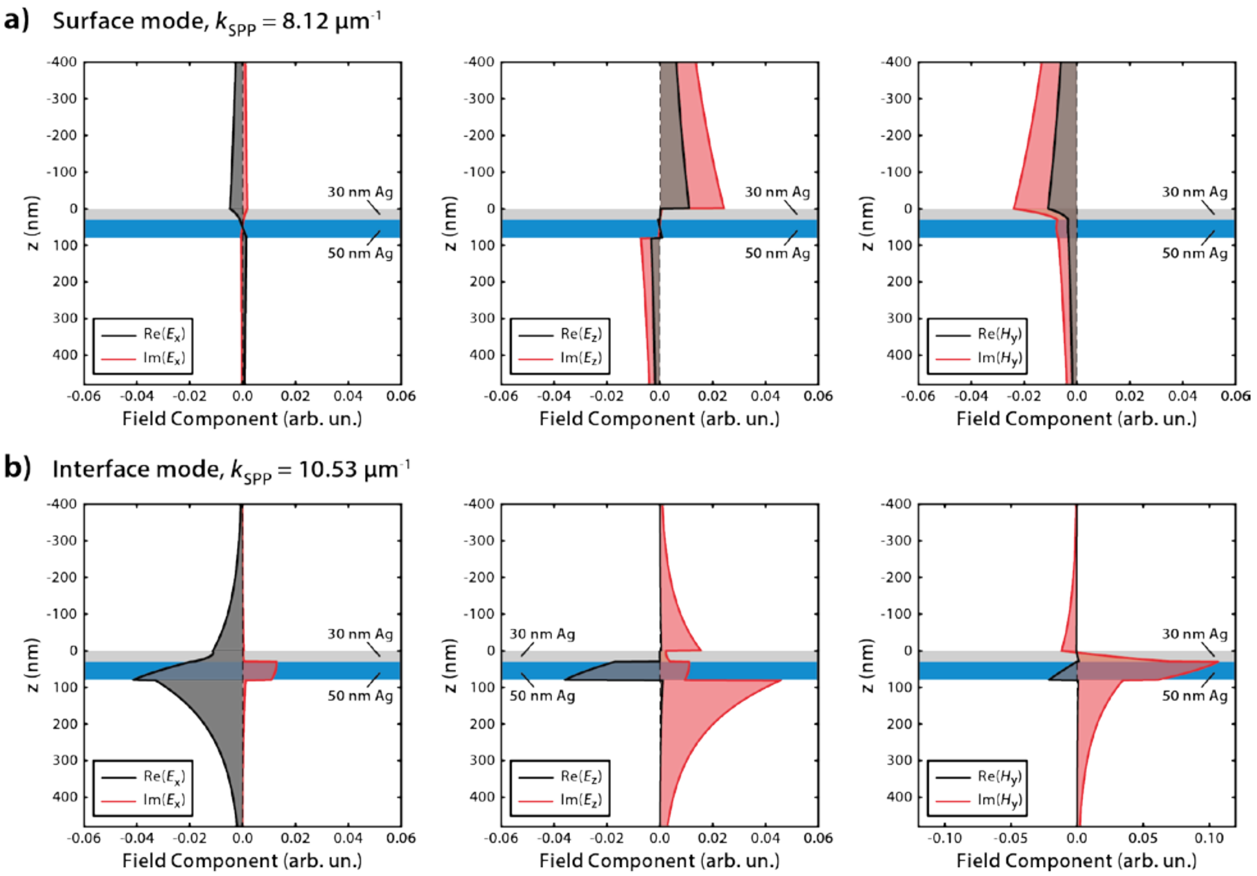

Supplementary Figure 4: Calculated plasmon mode profiles. Analytically calculated mode profiles for the two distinct plasmon modes identified in Figure 2d. The *z*-dependence (*z* corresponds to the direction perpendicular to the sample surface) of the relevant electromagnetic components for the surface (panel **(a)**) and buried interface (panel **(b)**) surface plasmon polariton (SPP) modes, excited at an energy of 1.577 eV, are plotted in relative arbitrary units.

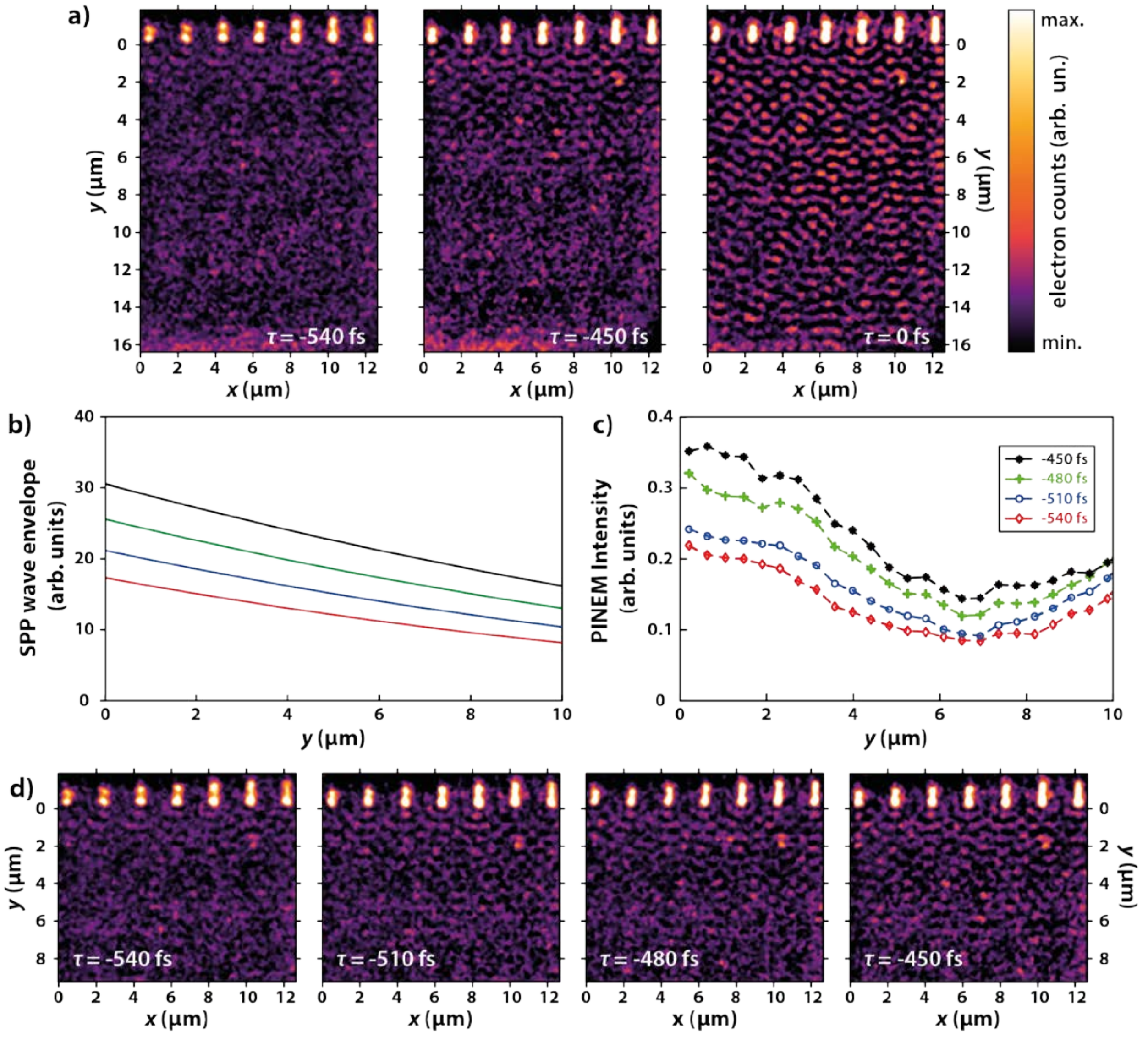

Supplementary Figure 5: Details of spatiotemporal PINEM imaging. (a) Real-space time-resolved photon-induced near-field electron microscopy (PINEM) images of the plasmonic near-field at the buried Ag/Si_3N_4 interface (still frames from Supplementary Movie 1 at time delays τ = -540, - 450 and 0 fs), visualizing the progressive propagation of the interferometric surface plasmon polariton (SPP) wave. The color scale holds for all PINEM images in the figure. **(b)** Theoretical SPP wave envelopes along the propagation axis *y* at time delays τ = -540, -510, -480 and -450 fs. Solid lines are based on calculated cross-correlation traces between the optical pump pulse and the delayed electron probe bunch (105 and 650 fs full-width-at-half-maximum (FWHM), respectively), converted to spatial SPP envelopes along *y* by assuming an SPP group velocity of $v_g = 1.0 \times 10^8$ m $s^{-1} \approx c/3$. The graph focuses on negative time delays that correspond to the temporal window in which the propagating SPP wave traverses the experimental observation window. **(c)** Experimental SPP wave intensity along the propagation axis *y* at time delays τ = -

540, -510, -480 and -450 fs. The SPP wave intensity at each point and delay is calculated as the integrated intensity of the SPP interference features in the spatial fast Fourier transform (FFT) of the corresponding spatial slice. The *y*-range of each of the slices corresponds to y = 0.42 μm. The relative magnitudes and overall trends of the experimental data match the theoretical expectation in panel b well. **(d)** Real-space PINEM images in the observation window at time delays τ = 540, -510, -480 and -450 fs, showing the evolution of the buried interferometric plasmonic wave in sequential frames 30 fs apart. Despite the convolution with a 650 fs FWHM electron bunch, the propagation characteristics of the SPP wave can be reliably extracted using the spatial Fourier analysis approach (Figure 3).

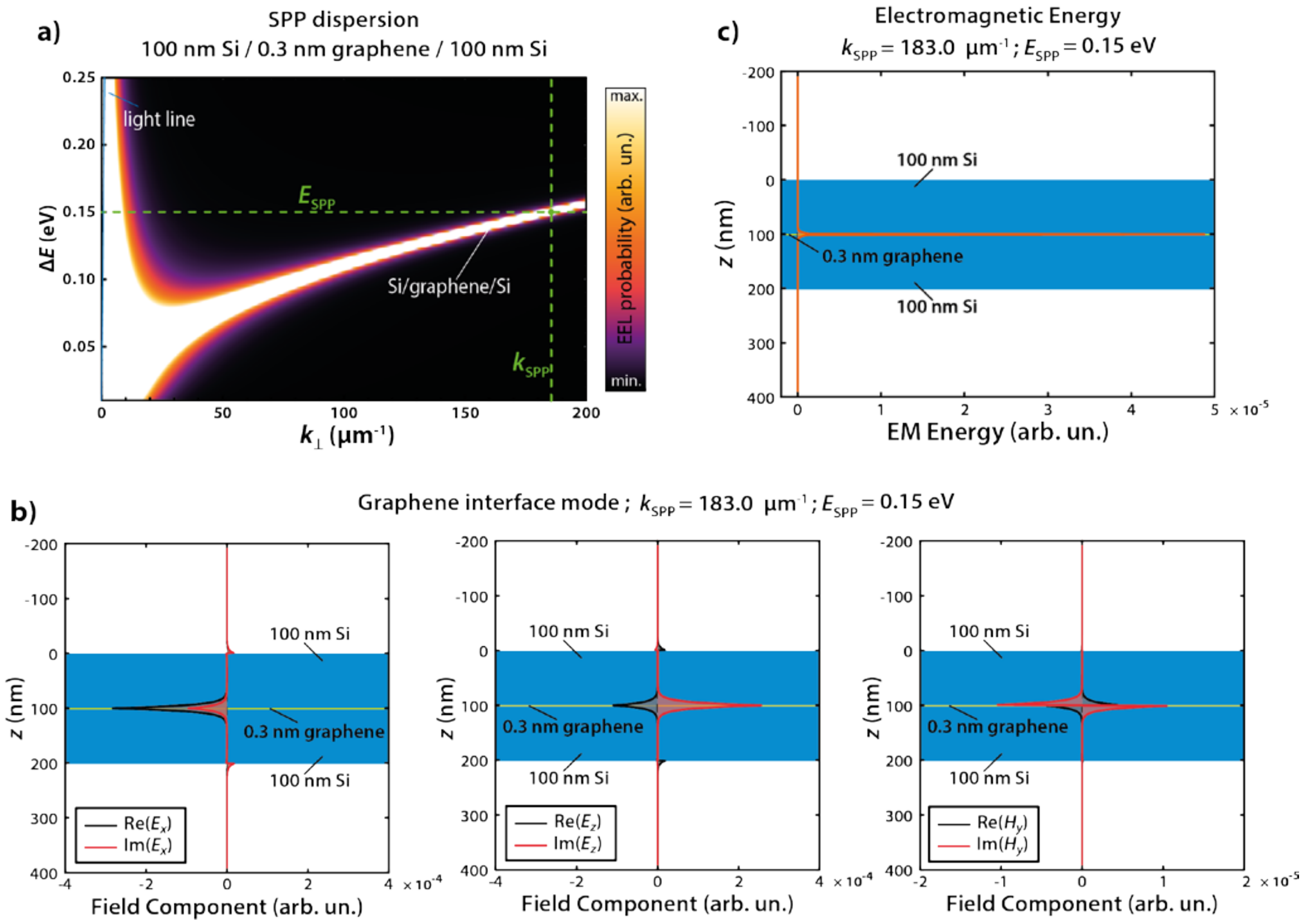

Supplementary Figure 6: Analytically modeling of the buried SPP mode in a Si/graphene/Si stack. (a) Analytically calculated electron energy loss (EEL) probability of 200 keV electrons traversing a 100 nm Si / 0.3 nm graphene / 100 nm Si material stack at normal incidence, as a function of energy loss ΔE and transversal momentum transfer $k_\perp$ (with respect to the beam direction). The dispersion branch corresponds to the buried surface plasmon polariton (SPP) mode propagating along the graphene layer. The light line is indicated by the solid straight line. **(b)** The z-dependence (z corresponds to the direction perpendicular to the sample surface) of the relevant electromagnetic field components for the interface SPP modes, at an energy $E_{SPP} = 0.15$ eV, and wavevector $k_{SPP} = 183$ μm^{-1}. Field components are plotted in relative arbitrary units. The vast majority of the electromagnetic energy (panel **(c)**) is carried by the electric field components. **(c)** Distribution of the electromagnetic energy of the buried SPP mode along the z-direction. The electromagnetic energy is strongly confined to the graphene layer, with 99.99% contained within the material stack.

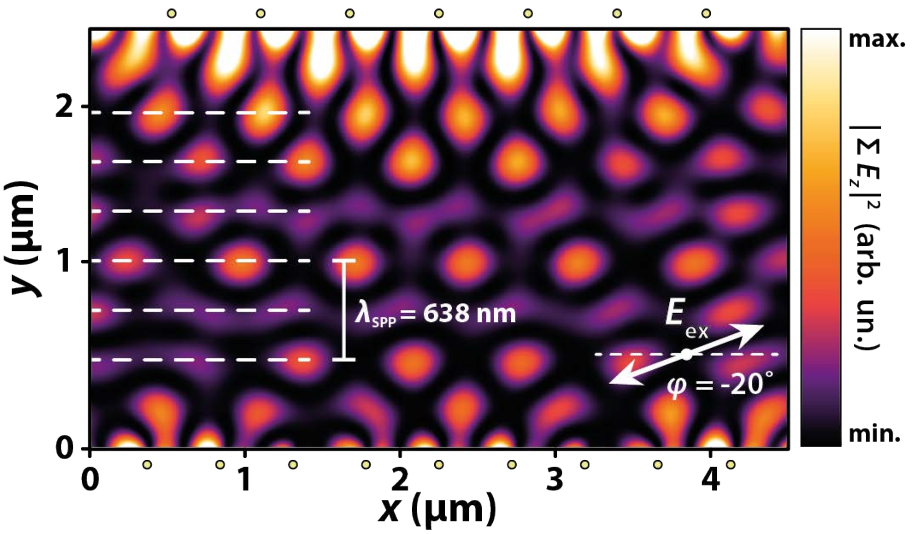

Supplementary Figure 7: Analytical SPP interference simulation. Analytically calculated plasmonic interference pattern based on simple point dipole surface plasmon polariton (SPP) sources (relative positions indicated by filled circles at panel top and bottom). Dipoles in each array are of equal strength, with their maximum radiated field oriented along the double-headed white arrow (i.e. the corresponding dipole oscillates along the direction perpendicular to the arrow). Plotted is the squared modulus of the linear superposition of E_z-components radiated by the different sources. Calculated with corresponding geometry and a carrier wavelength of λ_{SPP} = 638 nm, this simple dipole model shows excellent agreement with the experimental photon-induced near-field electron microscopy (PINEM) image of figure 4d, including the alternating pattern of continuous intensity wiggles, the arrays of intensity islands and the $\lambda_{SPP}/2$ periodicity along the y direction.

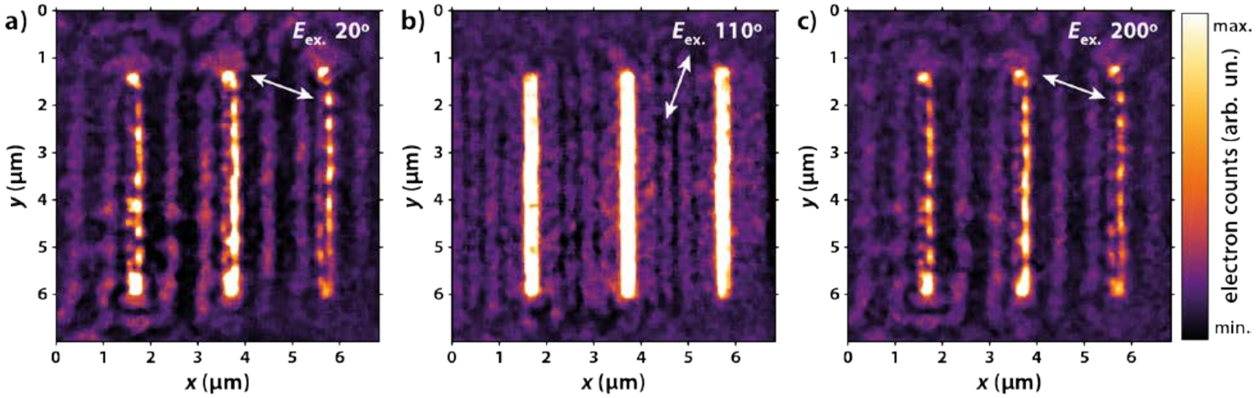

Supplementary Figure 8: Plasmonic transient grating experiments. Real space photon-induced near-field electron microscopy (PINEM) images of the polarization dependence of the plasmonic interference pattern at the buried Ag/Si_3N_4 interface for excitation polarization angles of **(a)** 20°, **(b)** 110° and **(c)** 200°. These still frames from Supplementary Movie 2 show the effective periodicity doubling of the transient plasmonic grating at equivalent polarization angles of 20 and 200°. The plasmons are photoinduced by a linearly polarized optical pump pulse (800 nm, 105 fs full-width-at-half-maximum (FWHM)), and are imaged at zero time delay using only electrons that had gained energy from the PINEM exchange interaction. The color scale holds for all images in the figure.

Supplementary References

1. Piazza, L. *et al.* Simultaneous observation of the quantization and the interference pattern of a plasmonic near-field. *Nat. Commun.* **6**, 6407 (2015).
